# Clinical features, outcome and HLA subtypes in Eastern patients with anti-IgLON5 disease: a multicenter study

**DOI:** 10.1186/s13023-026-04441-z

**Published:** 2026-06-12

**Authors:** Yining Gao, Xiaobo Sun, Yifan Zhou, Huoqing Luo, Lu He, You Ni, Huanyu Meng, Ying Zhang, Yaying Song, Ying Wang, Fan Song, Meiyuan Chen, Yajun Lian, Yuan Chen, Xing Zhao, Xiang Zhang, Xiangjun Chen, Guomin Xie, Cui Zhao, Xiaoming Zhou, Xiao Hu, Youming Long, Jinhua Qiu, Yong You, Zhongyan Zhao, Yi Li, Meihong Wang, Suya Sun, Zhifeng Mao, Qinming Zhou, Xiaofen Zhong, Sheng Chen

**Affiliations:** 1 Department of Neurology, Wuxi branch of Shanghai Ruijin Hospital, Wuxi, China; 2 Co-innovation Center of Neuroregeneration, Nantong University, Nantong, China; 3 Department of Neurology, The Third Affiliated Hospital of Sun Yat-Sen University, Guangzhou, China; 4 School of Life Science and Technology, ShanghaiTech University, Shanghai, China; 5 Department of Neurology, Renji Hospital, Shanghai Jiao Tong University School of Medicine, Shanghai, China; 6 Department of Neurology, The First Affiliated Hospital of Dalian Medical University, Liaoning, China; 7 Department of Neurology, The Affiliated Hospital of Hangzhou Normal University, Zhejiang, China; 8 Department of Neurology, The First Affiliated Hospital of Zhengzhou University, Zhengzhou, China; 9 Department of Neurology, Guangdong Second Provincial General Hospital, Guangzhou, China; 10 Department of Neurology, Huashan Hospital and Institute of Neurology, Fudan University, Shanghai, China; 11 Department of Neurology, Ningbo Medical Centre of Lihuili Hospital, Ningbo, China; 12 Department of Neurology, Pu’er People’s Hospital, Yunnan, China; 13 Department of Neurology, The People’s Hospital of Guizhou Province, Guizhou, China; 14 Department of Neurology, The Second Affiliated Hospital of Guangzhou Medical University, Guangzhou, China; 15 Department of Neurology, Huizhou Central People’s Hospital, Huizhou, China; 16 Department of Neurology, The Second Affiliated Hospital of Hainan Medical University, Haikou, China; 17 International Center for Aging and Cancer (ICAC), Hainan Medical University, Hainan, China; 18 Key Laboratory of Brain Science Research & Transformation In Tropical Environment Of Hainan Province, Hainan, China; 19 Department of Neurology, People’s Hospital of Hainan Province, Hainan, China; 20 Department of Neurology, Qilu Hospital of Shandong University, Shandong, China; 21 Department of Neurology, Yuxi City People’s Hospital, Yunnan, China; 22 Department of Clinical Medicine, Medical School, Xiangnan University, Chenzhou, China; 23 Department of Biotherapy Centre, The Third Affiliated Hospital of Sun Yat-sen University, Guangzhou, China; 24 Neuroimmunology Group, KingMed Diagnostic Laboratory, Guangzhou, China

**Keywords:** Anti-IgLON5 disease, Multicenter, Clinical characteristics, HLA-DQA1*01:05, Molecular dynamics simulations

## Abstract

**Background:**

Anti-IgLON5 disease, a rare autoimmune neurological disorder, remains understudied in Eastern populations. This study aimed to characterize the clinical characteristics, treatment responses, and long-term outcomes of Chinese patients with anti-IgLON5 disease in a multicenter Chinese cohort.

**Methods:**

This retrospective multicenter study enrolled 24 patients with anti-IgLON5 disease confirmed by serum and/or cerebrospinal fluid antibody testing from 17 centers in China. Human leukocyte antigen (HLA) typing was performed on patient blood samples. Clinical characteristics, mRS/ICS outcomes, treatment response, relapse, and long-term outcomes were analyzed. Short-term response was defined as improvement in mRS from admission to discharge, and relapse was defined as recurrence or worsening of symptoms after initial clinical improvement.

**Results:**

The mean age at onset was 59.6 years. Among 18 patients who received immunotherapy, 10/18 (55.6%) met the definition of achieving a short-term response. Responders included more women (6/10 vs. 2/8), fewer patients with bulbar symptoms (4/10 vs. 5/8), and more frequently had HLA-DRB1*10:01 or HLA-DQB1*05:01 (8/10 vs. 4/8) compared with non-responders. Men aged ≥ 65 years had poorer outcomes, whereas younger men and most women responded well. Long-term follow-up was performed on 16 patients (median 18 months, range 3–60 months), and eight withdrew from follow-up. Relapse occurred in 4/16 patients (25.0%). It typically occurred about 6–12 months after discharge, often following abrupt treatment withdrawal, and was effectively controlled with re-treatment. Kaplan–Meier analysis indicated that relapse risk was the highest within the first 6 months post-discharge. Early treatment (≤ 6 months) tended to be associated with more favorable outcomes. Overall, 10/14 immunotherapy-treated patients with long-term follow-up (71.4%) showed improvement, and 7/14 (50.0%) became asymptomatic. In this cohort, HLA-DQA1*01:05 and HLA-DRB1*10:01/HLA-DQB1*05:01 were over-represented among the typed patients, and the response rate to immunotherapy was numerically higher than that in the Western population (55.6% vs. 40%). The misdiagnosis rate was 33.3%. The median diagnostic delay was 2 months (IQR, 1–12 months; range, 2 days–6 years).

**Conclusions:**

As the largest cohort of anti-IgLON5 disease in China to date, this multicenter series demonstrated that Chinese and Western patients with anti-IgLON5 disease have both shared and distinct clinical characteristics. Chinese patients exhibited relatively favorable responses to early immunotherapy, relapse susceptibility requiring prolonged treatment, and potential HLA associations, offering new insight into disease management.

**Supplementary Information:**

The online version contains supplementary material available at 10.1186/s13023-026-04441-z.

## Background

The discovery of numerous autoantibodies has greatly advanced the understanding of autoimmune encephalitis (AE) [1, 2] and prompted a revision of its diagnostic approach. An antibody against IgLON family member 5 (IgLON5) was first identified in 2014 in eight patients presenting with sleep disturbances and sleep-disordered breathing [3]. Although the pathogenicity of anti-IgLON5 remains unclear, accumulating evidence suggests that this antibody can induce both autoimmunity and neurodegeneration, resulting in irreversible and long-lasting damage to the central nervous system (CNS) [4–6].

Anti-IgLON5 disease is an exceptionally rare autoimmune neurological disorder [7] with a clinical profile distinct from that of common autoimmune encephalitis (AE), primarily affecting adults aged ≥ 60 years [8]. Most patients with anti-IgLON5 disease exhibit sleep-related symptoms, sleep-disordered breathing, bulbar dysfunction, cognitive decline, and gait disturbances [9–11]. The disease often follows a chronic progressive course and may become life-threatening due to respiratory complications, severe bulbar involvement, and sudden clinical deterioration [3, 8, 12]. Patients often experience misdiagnosis and delayed diagnosis due to the heterogeneous and frequent atypical presentations of the disease, while the response to immunotherapy remains variable and incompletely understood [13–15]. A recent study identified a strong association between anti-IgLON5 disease and the human leukocyte antigen (HLA) alleles HLA-DRB1*10:01 and HLA-DQB1*05:01 [16]. Additionally, postmortem analyses revealed tauopathy and neuronal loss in most IgLON5-IgG-positive cases [17].

Although multicenter studies investigating the clinical characteristics of anti-IgLON5 disease have been conducted in other countries [8, 18], research conducted on Eastern populations remains limited. Specifically, it is unclear whether the HLA enrichment reported in Western anti-IgLON5-positive cohorts, especially HLA-DRB1*10:01 and HLA-DQB1*05:01, is also present in Chinese patients, whether additional co-segregating alleles such as HLA-DQA1*01:05 exist, and whether HLA subtypes may be related to phenotype, immunotherapy response, or relapse risk. This study presents the largest systematic and comprehensive multicenter investigation of the demographic characteristics, clinical manifestations, treatment outcomes, and prognoses of patients with anti-IgLON5 disease in China. By addressing these knowledge gaps and comparing the findings with Western cohorts, this study aims to clarify population-specific differences in patients with anti-IgLON5 disease while considering the potential influence of genetic background, healthcare pathways, follow-up duration, treatment strategies, and case mix.

## Methods

### Study design and main outcomes

This was a retrospective multicenter cohort study including patients from 17 centers in China between February 2017 and February 2025 (Supplementary Material). Cases were derived from a multicenter AE cohort and centrally screened for anti-IgLON5 antibodies. Those with anti-IgLON5 antibodies who met the inclusion criteria were included in the study. The main outcomes of interest were short-term functional response at discharge, long-term functional outcome at the last follow-up, and relapse during follow-up. The secondary exploratory endpoints included ICS (anti-IgLON5 disease composite score) changes, HLA profiles, and HLA-related analyses.

### Patients

**Fig. 1:**
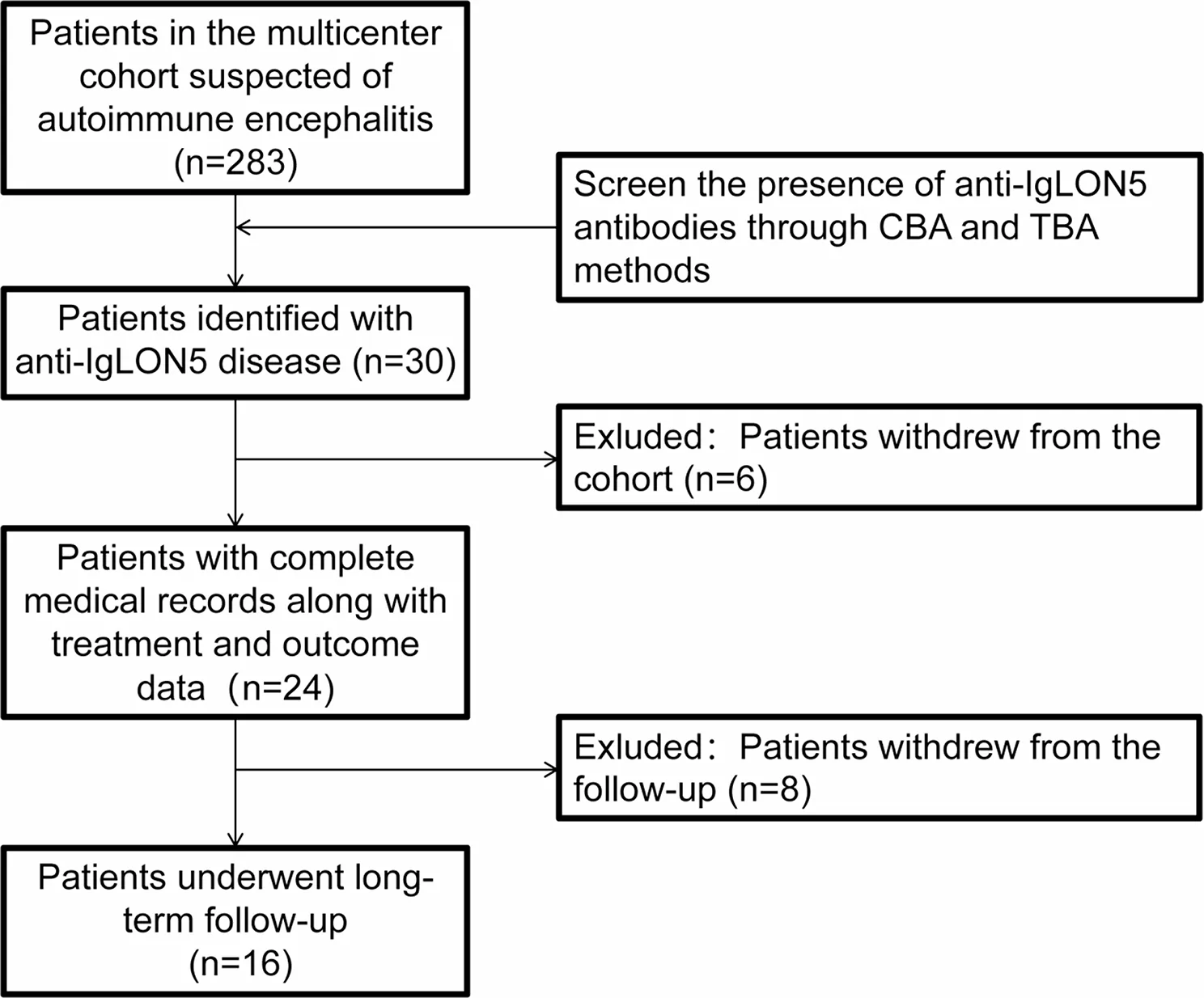
Flowchart of patient inclusion for the study

A total of 283 patients with suspected AE were identified and enrolled in the multicenter cohort. February 2025 served as the data cutoff time for the present analysis. Suspected AE referred to patients with clinical manifestations suggestive of AE in routine practice, including sleep disorders, cognitive impairment, movement disorders, bulbar symptoms, seizures, psychiatric symptoms, and other unexplained CNS manifestations. Serum or cerebrospinal fluid (CSF) samples collected from these patients were screened for anti-IgLON5 antibodies at KingMed Diagnostic Laboratory (Guangzhou, China) using both cell-based assay (CBA) and tissue-based assay (TBA) methods. Patients with a compatible clinical syndrome and positive anti-IgLON5 antibodies in serum and/or CSF were included in the study. Patients were excluded if an alternative diagnosis better explained the presentation, including infection, metabolic/toxic causes, and other definite autoimmune/paraneoplastic disorders. Finally, 24 patients diagnosed with AE and positive for anti-IgLON5 antibodies in either serum or CSF were included [1] (Fig. 1). These patients were recruited from 17 centers. Eight of these patients were previously described in another study [19].

Patients were followed up at 3 months, 6 months, and every 6 months thereafter after discharge, either by telephone or outpatient visits according to patient preference. Data quality was ensured through double-entry verification and periodic cross-checking by two senior neurologists. Any discrepancies were resolved through discussion with local investigators. Anti-IgLON5 antibody testing was performed centrally at the KingMed Diagnostic Laboratory, and clinical data were collected using the same predefined variables, including mRS (the Modified Rankin Scale) and ICS assessments, to improve inter-center comparability. 

Of the initially identified patients, six withdrew before inclusion in the final cohort. Among the 24 included patients, eight were lost to long-term follow-up. The retrospective multicenter design precluded obtaining the specific reasons for withdrawal/loss to follow-up, as they were not systematically recorded and could not be reliably retrieved. Follow-up completeness was assessed according to the availability of longitudinal post-discharge outcome data.

### Antibody and gene assays

Antibody detection was performed using the CBA method (KingMed Diagnostics Co., Ltd., China) [19]. Briefly, monospecific testing was performed using IgLON5-transfected HEK cells, with green fluorescent protein (GFP) as an internal reference. Serum samples were routinely diluted 1:10, whereas CSF samples were used undiluted. Specific immunofluorescence observed in transfected cells indicated a positive serum sample. If a positive signal was observed, serial 10-fold dilutions were performed to determine the endpoint titer.

**Fig. 2:**
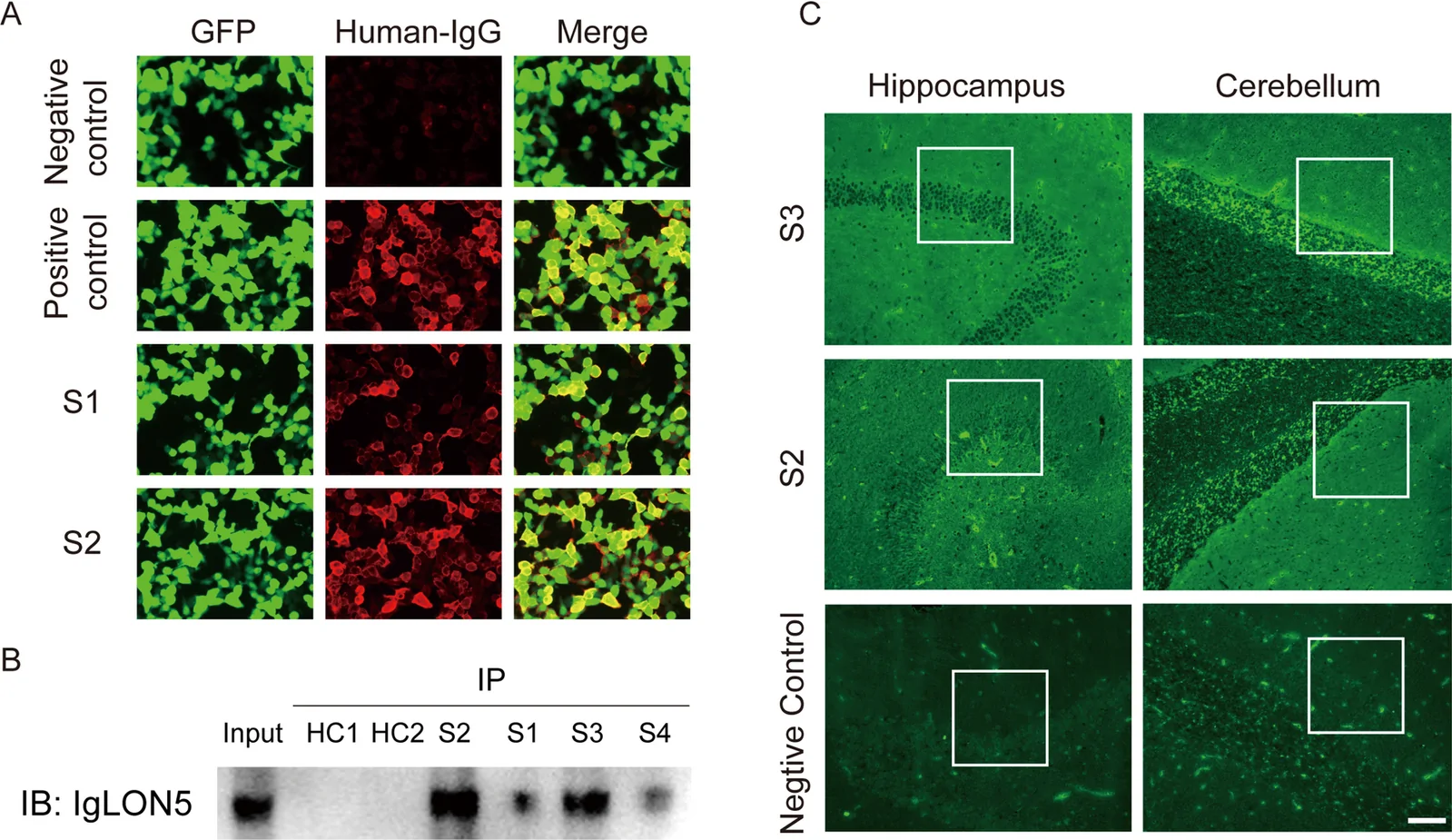
Detection of anti-IgLON5 antibodies in serum and cerebrospinal fluid. (**A**) CBA images, showing varying antibody titers (S1, 1:30; S2, 1:300) with negative and positive serum controls. Monospecific testing used IgLON5-transfected HEK cells, with green fluorescent protein (GFP) as an internal reference to distinguish transfected cells. (**B**) Four blood samples representing different antibody levels (S2, 1:300; S1, 1:30; S3, 1:100; S4, 1:30) were compared using co-immunoprecipitation, the gold standard, for quality control (HC = healthy control). (**C**) Fluorescence of the hippocampal neuropil and cerebellum, showing patchy staining in the cerebellar stratum granulosum

Four blood samples with different antibody levels (S1, 1:30; S2, 1:300; S3, 1:100; and S4, 1:30) (Fig. 2A) were compared as quality controls using the co-immunoprecipitation gold standard (Fig. 2B). TBA analysis was conducted on monkey brain slices to examine IgLON5 antibody reactivity patterns in the hippocampus and cerebellum (Fig. 2C). Undiluted CSF was incubated on the tissues for 2 h at room temperature, followed by washing in phosphate-buffered saline (PBS)-0.1% Tween-20 and incubating with fluorescein-conjugated goat anti-human IgG. Fluorescence patterns were evaluated using an immunofluorescence microscope. Detailed TBA and immunoprecipitation procedures are provided in the Supplementary Material.

HLA alleles were identified in blood samples as described previously [20]. Serum and CSF samples were screened against autoantibody panels associated with AE, CNS inflammatory demyelinating diseases, paraneoplastic syndromes, and autoimmune rheumatic diseases. 

### Therapeutic interventions and outcome assessments

The efficacy of short-term (intravenous methylprednisolone [IVMP], intravenous immunoglobulin [IVIG], or plasma exchange) and long-term (oral corticosteroids, azathioprine, or mycophenolate mofetil) immunotherapies in the patients was evaluated.

Patients were assessed using ICS and mRS scores at admission and discharge [21]. ΔmRS was defined as the admission mRS score minus the discharge mRS score, and ΔmRS′ was defined as the admission mRS score minus the last follow-up mRS score. Similarly, ΔICS was defined as the admission ICS minus the discharge ICS, and ΔICS′ was defined as the admission ICS minus the last follow-up ICS. Patients were classified as short-term responders if their mRS score improved at discharge compared with the admission score (ΔmRS > 0), and as non-responders if the discharge mRS score was unchanged or worse (ΔmRS ≤ 0).

### AlphaFold prediction

The AlphaFold model was employed and specifically trained for multimeric inputs with known stoichiometry, referred to as the AlphaFold multimer. This approach significantly improves the accuracy of predicted multimeric interfaces compared with the input-adapted single-chain AlphaFold model and maintains high intra-chain accuracy. Therefore, AlphaFold multimer was used to predict the complex structure of HLA-DQA1*01:05 and IgLON5 based on their peptide sequences. The predicted complex was subsequently subjected to molecular dynamics (MD) simulation [22].

### MD simulations

The Desmond suite of Schrödinger LLC (version 2021-1, D. E. Shaw Research, New York, NY, USA) was used to perform MD simulations of the complexes to investigate the binding stability and specificity of the compounds. The simulation systems were prepared by applying an OPLS4 force field to the complexes, and an orthorhombic box was generated with a 10 Å buffer around each complex. Simple-point-charge water molecules were added to fill the box, and the system was neutralized with 0.15 M NaCl, with additional Na⁺ and Cl⁻ ions distributed randomly. The model system was relaxed before the simulation. Temperature and pressure in the NPT ensemble were maintained at 300 K and 1 atm, respectively. Following equilibration, MD simulations were run for 1000 ns [23].

### Statistical analysis

Statistical analyses were conducted using GraphPad Prism version 8.0. Categorical variables were compared using Fisher’s exact test or the chi-squared test, as appropriate. Continuous variables are presented as mean ± standard deviation (SD) when approximately normally distributed, and as median with interquartile range (IQR) or range when non-normally distributed. Ordinal or non-normally distributed variables, including antibody titers, were analyzed using non-parametric methods, such as the Mann–Whitney U test. The Kaplan–Meier method was used to estimate relapse-free survival. Subgroup analyses were performed according to sex, age, HLA status, CSF antibody status, and treatment timing as exploratory analyses. For selected key exploratory subgroup comparisons, effect sizes were reported as odds ratios (ORs) with 95% confidence intervals (CIs) where appropriate and estimable. Missing data were managed using an available-case approach; patients with missing values for a given variable were excluded only from the corresponding analysis, and no imputation was performed. The availability of key variables varied across analyses, including HLA typing (19/24), extended HLA typing (10/24), treatment data (20/24), discharge mRS scores (20/24), and long-term follow-up data (16/24). No formal interaction testing, no formal multivariable adjustment for potential confounders, and no correction for multiple comparisons were performed due to the limited sample size. All statistical tests were two-tailed, and a p-value of < 0.05 was considered statistically significant

## Results

### Demographic characteristics

The mean age of the 24 enrolled patients was 59.6 years (range, 33–76 years), with a male-to-female ratio of 1.4. The median time from symptom onset to diagnosis was 2 months (IQR, 1–12 months; range, 2 days–6 years). Notably, 33.3% of the patients (8/24) were initially misdiagnosed as having other conditions, including Parkinson’s disease, chorea, progressive supranuclear palsy, viral encephalitis, restless legs syndrome, and epilepsy.

### Clinical symptoms

The observed clinical symptoms are summarized in Table 1. Sleep disorders were the most common symptom, observed in 17 patients (70.8%). Cognitive impairment occurred in 14 patients (58.3%), manifesting as impaired orientation, diminished calculation ability, amnesia, and poor logical reasoning. Movement disorders were observed in 14/24 patients (58.3%), including gait instability (6/24, 25.0%), tremor (5/24, 20.8%), bradykinesia (2/24, 8.3%), dystonia (2/24, 8.3%), stereotypic movements (1/24, 4.2%), and tics (1/24, 4.2%), with some patients exhibiting multiple symptoms.

**Table 1:** Clinical characteristics of the patients

Characteristics	Value
**Sex**	
male	14/24 (58.3%)
female	10/24 (41.7%)
**Age (y)**	
average (range)	59.6 (33–76)
**Time span for diagnosis**	
median (IQR; range)	2 m (1–12 m; 2 d–6 y)
**Misdiagnosis**	8/24 (33.3%)
**Cardinal symptom**	
Sleep disturbances	17/24 (70.8%)
Cognitive impairments	14/24 (58.3%)
Movement disorder	14/24 (58.3%)
Gait instability	6/24 (25.0%)
Tremor	5/24 (20.8%)
Bradykinesia	2/24 (8.3%)
Dystonia	2/24 (8.3%)
Stereotypic movements	1/24 (4.2%)
Tics	1/24 (4.2%)
Neuropsychiatric symptoms	8/24 (33.3%)
Bulbar symptoms	12/24 (50.0%)
Dysphagia	8/24 (33.3%)
Central hypoventilation	3/24 (12.5%)
Dysarthria	2/24 (8.3%)
Dysautonomia	8/24 (33.3%)
Seizures	4/24 (16.7%)
Headache	2/24 (8.3%)
Dizziness	2/24 (8.3%)
Diplopia	3/24 (12.5%)
**ICS scores (average)**	
At onset	8.5
At discharge	6.1
ΔICS	2.7
**Antibody status**	
Positive in CSF	19/24 (79.2%)
Median titer in CSF	1:100
Positive in serum	24/24 (100%)
Median titer in serum	1:300
**CSF analysis**	
Pleocytosis	4/24 (16.7%)
Elevated protein level	8/24 (33.3%)
HLA-DRB1*10:01; DQB1*05:01, n(%)	15/19 (78.9%)

ICS: anti-IgLON5 disease composite score  CSF pleocytosis was defined as white blood cell (WBC) count ≥ 5/μL, and an elevated protein level was defined as ≥ 450 mg/L  Percentages for symptom subcategories were calculated using the full cohort (N = 24) as the denominator

Bulbar symptoms were present in 12/24 patients (50.0%), including dysphagia (8/24, 33.3%), central hypoventilation (3/24, 12.5%), and dysarthria (2/24, 8.3%). Neuropsychiatric symptoms and dysautonomia were each reported in eight patients (33.3%). Seizures occurred in four patients (16.7%), and diplopia in three (12.5%). Additionally, two patients reported headaches (8.3%), and two experienced dizziness (8.3%). The mean ICS of the patients was 8.5.

A subset of ten patients had a history of prior infection, tumor, or other immune-related conditions. Specifically, an infectious prodrome was observed in eight cases. One patient had an autoimmune disorder and tested positive for anti-thyroid-peroxidase and anti-thyroglobulin antibodies. Another patient was diagnosed with kidney cancer, presenting primarily with psychiatric and behavioral abnormalities, bulbar symptoms, sleep disturbances, and tremor.

### Neuroimaging Results

Magnetic resonance imaging (MRI) mostly revealed non-specific findings, with non-specific brain atrophy or ischemic lesions commonly observed in elderly patients. However, one 50-year-old female patient exhibited age-inconsistent brain atrophy, with marked atrophy in the cerebellum, midbrain, and occipital lobes.

**Fig. 3:**
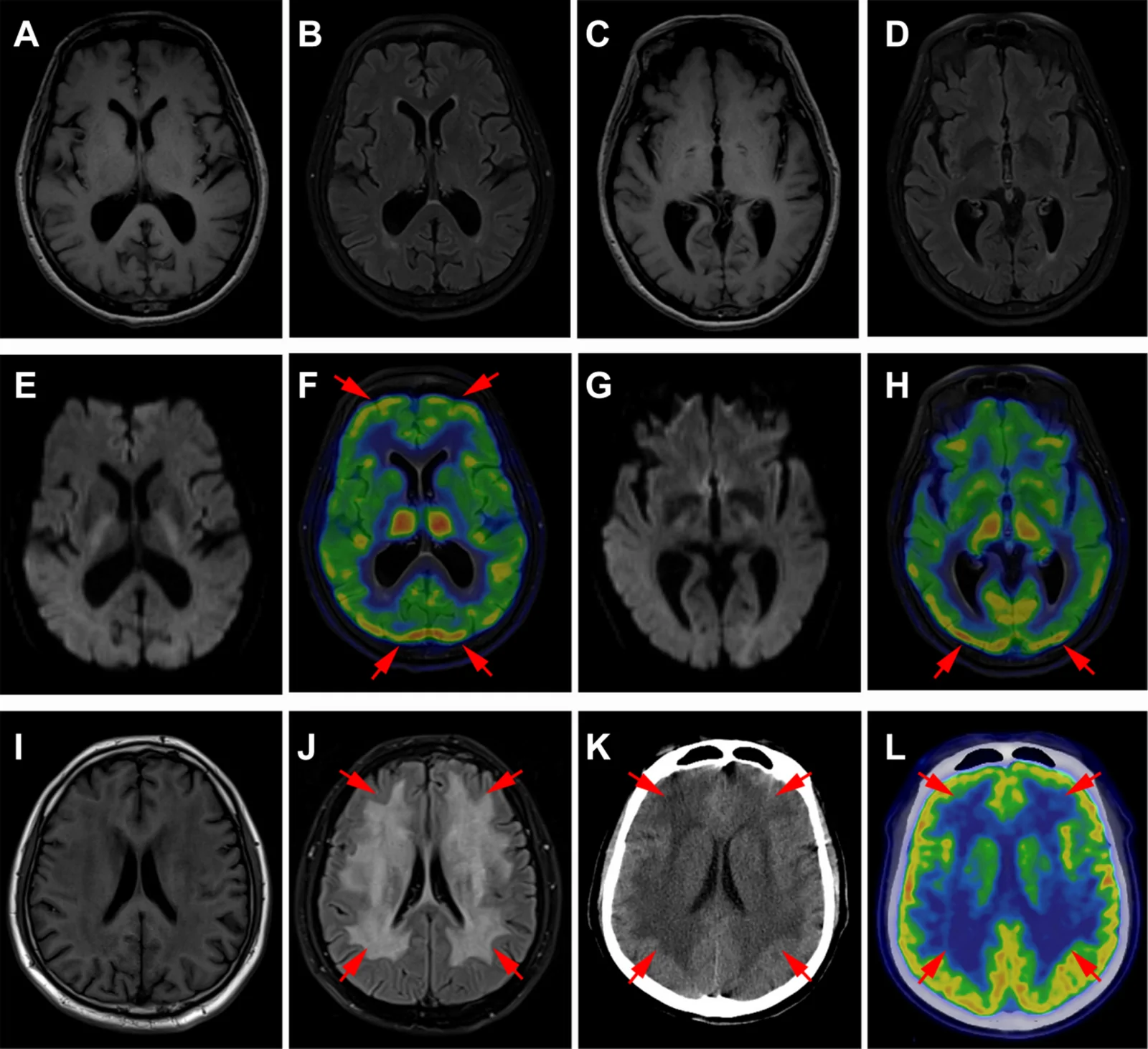
Imaging results of patients 2 and 3. (**A**–**H**) ^18^F-DPA-714 PET/MRI of Patient 3, showing increased tracer uptake in the frontal and occipital lobes (**F** and **H**), with no obvious abnormalities on T1 (**A** and **C**), T2-FLAIR (**B** and **D**), or diffusion-weighted imaging (**E** and **G**). (**I**–**J**) Brain MRI of Patient 2, demonstrating slightly low T1 signal and significantly high T2-FLAIR signal in the bilateral white matter. (**K**–**L**) ^18^F-FDG PET/CT of Patient 2, showing extensive low-density on CT (**K**) and diffuse reduction in ^18^F-FDG uptake in the bilateral white matter (**L**)

MRI showed encephalitis-like changes in three patients. A 51-year-old male patient showed a high T2 signal in the medial temporal lobe with post-contrast enhancement. A 38-year-old female showed strip-like enhancement in the left frontal and parietal sulci, and a 33-year-old male displayed focal cortical swelling in the right frontal lobe. Three patients underwent ^18^F-FDG positron emission tomography (PET)/computed tomography (CT) scans. ^18^F-FDG was used as a radiotracer for PET/CT. These scans revealed reduced temporal-lobe metabolism in two cases, and one patient showed diffuse reduction in ^18^F-FDG uptake in the white matter. One patient underwent ^18^F-DPA-714 PET/MRI to visualize microglial activation [24], which demonstrated increased metabolism in the frontal and occipital lobes (Fig. 3).

### Laboratory results and HLA findings in patients with anti-IgLON5 disease

All 24 patients tested positive for serum IgLON5-IgG, with a median titer of 1:300 (range, 1:30–1:1000). Among the 24 patients who underwent CSF testing, 19 (79.2%) tested positive for CSF IgLON5-IgG, with a median titer of 1:100 (range, 1:10–1:300). Abnormal CSF findings were detected in 33.3% (8/24) of the patients, including pleocytosis in four (50.0%; 28–100 cells/µL) and elevated protein levels in all eight (100.0%; 0.54–1.53 g/L).

Nineteen patients underwent HLA typing for the HLA-DRB1*10:01 and HLA-DQB1*05:01 alleles. Fifteen were found (78.9%) to have HLA-DRB1*10:01 or HLA-DQB1*05:01. Serum antibody titers were analyzed as ordinal data using the Mann–Whitney U test. Although allele carriers tended to have higher serum antibody titers than non-carriers, the difference was not statistically significant (*p* = 0.147).

Among these 19 patients, 10 underwent more extensive HLA typing. In this subgroup, all six patients with HLA-DRB1*10:01 and HLA-DQB1*05:01 also carried HLA-DQA1*01:05, whereas patients negative for HLA-DRB1*10:01 and HLA-DQB1*05:01 did not have the HLA-DQA1*01:05 allele (Table 2). The HLA-typed subgroups were defined by the testing performed and did not correspond directly to the 16 patients with long-term follow-up. All the six patients positive for HLA-DQA1*01:05 experienced sleep disturbances (100%). Four of them (66.7%) experienced fevers prior to disease onset, suggesting a possible infectious trigger, and one (16.7%) was seropositive for antibodies only.

**Table 2:** HLA alleles in the patients

Patients number	HLA-DQA1	HLA-DQA1	HLA-DQB1	HLA-DQB1	HLA-DRB1	HLA-DRB1
1	DQA1*01:02	DQA1*01:05	DQB1*05:01	DQB1*06:09	DRB1*10:01	DRB1*13:02
20	DQA1*02:01	DQA1*01:05	DQB1*05:01	DQB1*02:02	DRB1*10:01	DRB1*07:01
6	DQA1*01:03	DQA1*01:05	DQB1*05:01	DQB1*06:01	DRB1*10:01	DRB1*08:03
10	DQA1*01:04	DQA1*01:05	DQB1*05:01	DQB1*05:02	DRB1*10:01	DRB1*14:54
12	DQA1*01:02	DQA1*01:05	DQB1*05:01	DQB1*05:02	DRB1*10:01	DRB1*15:01
3	DQA1*02:01	DQA1*01:05	DQB1*05:01	DQB1*02:02	DRB1*10:01	DRB1*07:01
24	DQA1*01:01	DQA1*05:08	DQB1*05:01	DQB1*03:01	DRB1*01:01	DRB1*12:01
22	DQA1*01:02	DQA1*01:04	DQB1*05:02	DQB1*05:03	DRB1*14:04	DRB1*16:02
5	DQA1*01:03	DQA1*05:01	DQB1*06:01	DQB1*02:01	DRB1*03:01	DRB1*08:03
2	DQA1*01:02	DQA1*01:02	DQB1*06:01	DQB1*06:02	DRB1*15:01	DRB1*15:01

ND: undetected

### Therapeutic interventions and outcomes

**Fig. 4:**
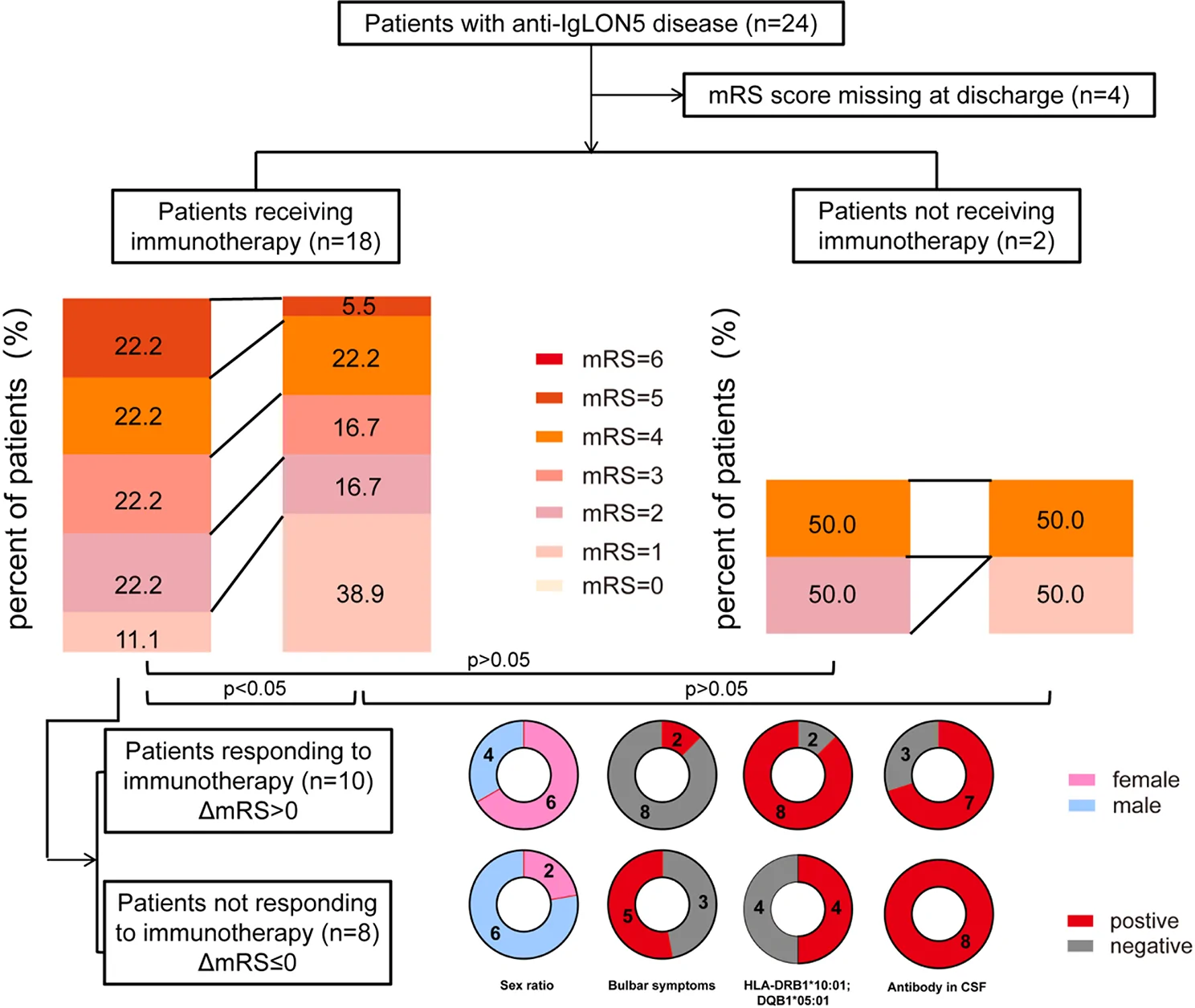
Immunotherapy efficacy. Left and right: Changes in Modified Rankin Scale (mRS) scores from admission to discharge in patients receiving immunotherapy and those not receiving immunotherapy, respectively. A significant decrease in mRS scores for patients receiving immunotherapy (p < 0.05). Bottom: Comparison of clinical characteristics between patients with effective responses to immunotherapy and those with ineffective responses

Treatment data were available for 20 patients, of whom 18 received immunotherapy, and two declined treatment. The acute immunotherapy regimens, time from onset to treatment, documented maintenance immunosuppressive therapy, and short-term response rates among immunotherapy-treated patients are summarized in Table 3. Patients who received immunotherapy demonstrated significantly lower mRS scores at discharge compared to admission (*p* < 0.05). Eight patients, all older than 53 years, did not respond to immunotherapy or experienced clinical deterioration. The 10 responders included more women (6/10 vs. 2/8; odds ratio (OR) 4.50, 95% confidence interval (CI) 0.59–34.61), fewer patients with bulbar symptoms (4/10 vs. 5/8; OR 0.40, 95% CI 0.06–2.70), and more frequently had HLA-DRB1*10:01 or HLA-DQB1*05:01 (8/10 vs. 4/8; OR 4.00, 95% CI 0.50–31.98) compared with the eight non-responders (Fig. 4).

**Table 3:** Treatment stratification, time from onset to treatment, and short-term response among immunotherapy-treated patients

Acute regimen	IVIG only	IVMP only	IVIG+IVMP	PE+IVIG +IVMP
No. of patients	3	5	7	3
Time from onset to treatment, median months (range)	12.0 (2.0–36.0)	1.0 (0.57–2.0)	1.0 (0.07–72.0)	0.58 (0.17–1.00)
Maintenance immunosuppressive therapy, n/N (%)	0/3 (0.0)	2/5 (40.0)	4/7 (57.1)	1/3 (33.3)
Responders at discharge, n/N (%)	1/3 (33.3)	1/5 (20.0)	5/7 (71.4)	3/3 (100.0)

Responders were defined as patients with improved mRS scores at discharge compared with admission (ΔmRS > 0)

### Follow-up results

Sixteen patients underwent long-term follow-up, with durations ranging from 3 to 60 months (median, 18 months; mean, 21.4 months), corresponding to a total follow-up time of 342 person-months (28.5 person-years). Among these, the median mRS score changed from 3.0 (IQR, 2.0–4.0) at admission to 2.0 (IQR, 1.0–4.0) at discharge and 1.0 (IQR, 0–4.5) at the last follow-up. The corresponding median ICS changed from 7.5 (IQR, 4.75–9.25) to 4.0 (IQR, 2.0–8.5) and 1.5 (IQR, 0–14.0), respectively. Of the 14 immunotherapy-treated patients, five received IVMP as first-line therapy, one received IVIG, six were treated with a combination of IVIG and IVMP, and two received IVMP, IVIG, and plasma exchange. Overall, among the 14 immunotherapy-treated patients with long-term follow-up, seven (50.0%) achieved complete recovery and three (21.4%) showed partial improvement, for an overall improvement rate of 10/14 (71.4%). Four patients (28.6%) died during follow-up, three males within one year of disease onset and one female 18 months after onset.

Regarding symptom outcomes, sleep disturbance, gait abnormalities, and bulbar symptoms were the most frequent initial manifestations and showed the most notable improvements following immunotherapy, particularly in patients treated with IVIG or combined IVMP and IVIG. Cognitive impairment and dysautonomia improved more gradually, and residual sleep disturbance persisted in three patients despite general clinical improvement. All patients who improved or recovered received long-term immunotherapy with low-dose corticosteroids.

**Fig. 5:**
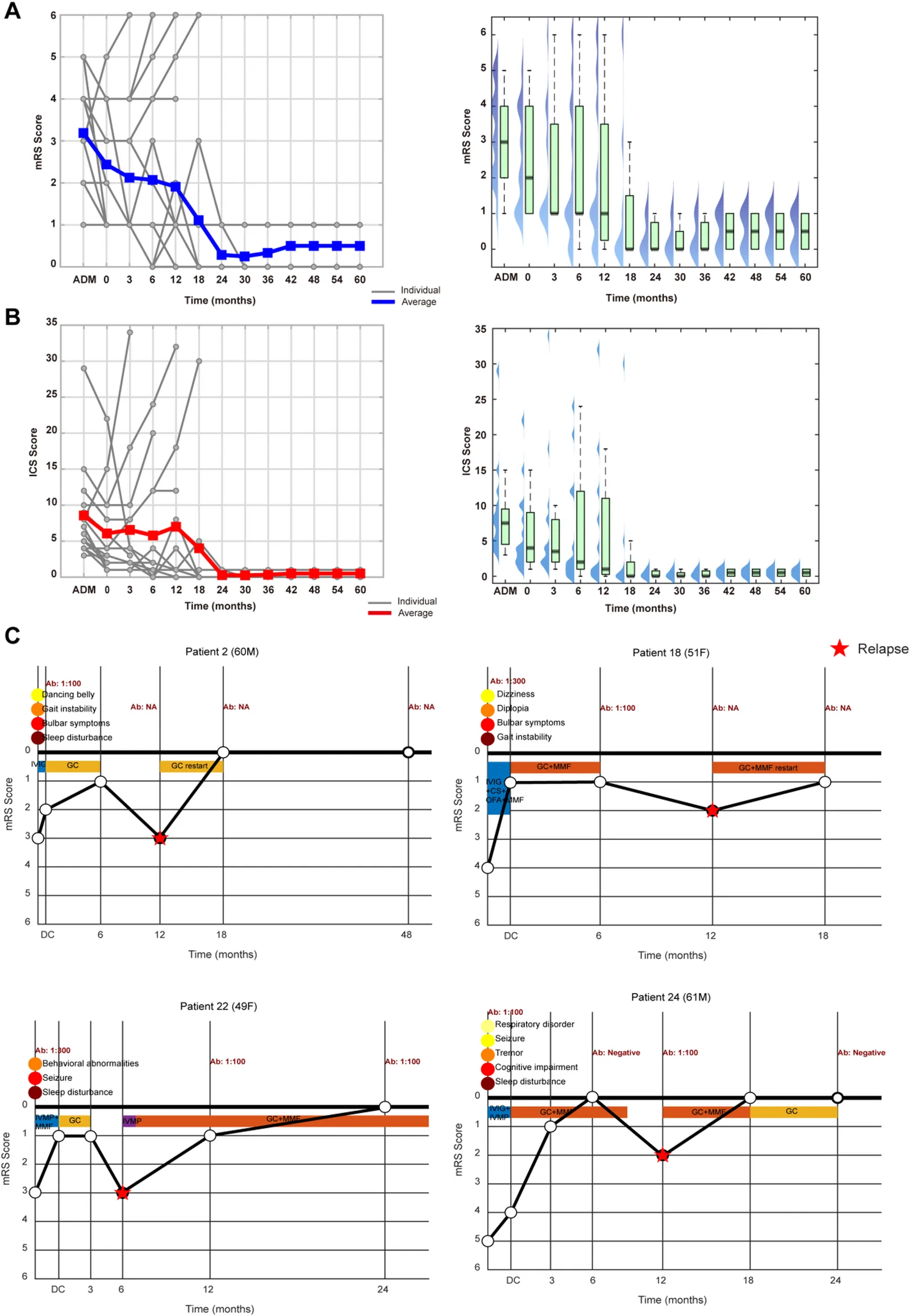
Follow-up timeline and relapse patterns. (**A** and **B**) Line and box plots of mRS (**A**) and ICS (**B**) scores in 16 patients from admission to 60-month follow-up. (**C**) Timeline from discharge to last follow-up for the four patients who experienced relapse (ADM, admission; DC, discharge; OFA, ofatumumab; IVMP, intravenous methylprednisolone; GC, glucocorticoid; MMF, mycophenolate mofetil)

Of the two patients who did not receive immunotherapy, Patient 15, a 36-year-old female, showed no improvement after one year of follow-up (mRS = 4), and Patient 20 (male) experienced partial improvement with symptomatic treatment, with mRS scores improving from 2 to 1, and remaining stable over 6 months (Figs. 5A and B).

Three patients tested positive for serum IgLON5 antibodies only and had HLA-DRB1*10:01 or HLA-DQB1*05:01 alleles. Among them, Patients 23 and 24, with initial serum antibody titers of 1:30 and 1:100, respectively, became seronegative after long-term follow-up following symptom resolution.

Notably, four patients (25.0%) who initially responded to immunotherapy experienced relapses after treatment discontinuation (Fig. 5C). Patient 2, a 60-year-old male, improved after IVIG treatment at disease onset. His initial symptoms included sleep disturbances, bulbar symptoms, gait instability, and a “dancing belly”-like movement. He received oral corticosteroids for 6 months post-discharge, relapsed 6 months after discontinuation, resumed corticosteroids, and became asymptomatic a year later. No relapse occurred during the subsequent 2-year follow-up. Patient 18, a 51-year-old female, showed significant improvement after receiving IVIG, corticosteroids, ofatumumab, and mycophenolate mofetil. Her initial symptoms included gait instability, bulbar symptoms, diplopia, and dizziness. She maintained oral corticosteroids and mycophenolate mofetil use post-discharge, became asymptomatic within 6 months, and her serum antibody titer decreased from 1:300 to 1:100. She relapsed one year later following a surgical procedure and was treated again with corticosteroids and mycophenolate mofetil. She regained an asymptomatic state within 6 months after resuming therapy. Patient 22, a 49-year-old female, improved after methylprednisolone pulse therapy and mycophenolate mofetil. Her initial symptoms included sleep disturbances, seizures, and behavioral abnormalities. After discharge, she continued oral corticosteroid therapy for 3 months and discontinued the medication after significant symptom improvement. Three months after stopping the medication, the patient experienced a relapse and received methylprednisolone pulse therapy. Her symptoms fully resolved one year later, and her follow-up antibody titers remained at 1:100. Patient 24, a 61-year-old male with sleep disturbances, cognitive impairment, bradykinesia, tremor, seizures, a respiratory disorder, and consciousness disturbance, received IVIG and corticosteroids during hospitalization, followed by one year of maintenance therapy with low-dose oral corticosteroids and mycophenolate mofetil. His symptoms improved, and he became seronegative (baseline titer, 1:100). However, he experienced a relapse after discontinuing treatment for 3 months, with the antibody titer rebounding to 1:100. He resumed therapy for 6 months and consequently achieved symptom resolution. Corticosteroids were then discontinued, and only mycophenolate mofetil administration was maintained. Repeat antibody testing performed after an additional 6 months was negative; thus, mycophenolate mofetil administration was discontinued. The patient experienced no relapse during a 6-month follow-up.

Two other patients underwent serum antibody testing after symptomatic improvement. A 38-year-old female became completely asymptomatic after 6 months of immunotherapy (baseline serum titer, 1:30; CSF negative) and remained seronegative 2 years later. A 70-year-old female improved after immunotherapy, although her sleep disturbances persisted. Her baseline serum titer was 1:1000, CSF titer was 1:100, and serum antibody titer remained at 1:100 over 5 years.

The seven male patients who improved and remained stable after immunotherapy during long-term follow-up were significantly younger than the three male patients who died within one year (mean age, 58.1 vs. 70.0 years; *p* < 0.01).

### Prognostic impact of treatment timing

We compared functional improvements (ΔmRS′) in patients who initiated immunotherapy within 6 months of disease onset and those treated later (early- and late-treatment groups, respectively). Among treated patients with available longitudinal mRS score data (*n* = 16), the early-treatment group (*n* = 12) demonstrated a higher mean ΔmRS′ than the late-treatment group (*n* = 4) (1.42 ± 2.57 vs. 0.50 ± 2.08, *p* > 0.05). Similarly, the early-treatment group showed a mean ΔICS′ of 1.33 ± 14.19 compared with − 3.75 ± 11.50 in the late-treatment group (*p* > 0.05).

**Fig. 6:**
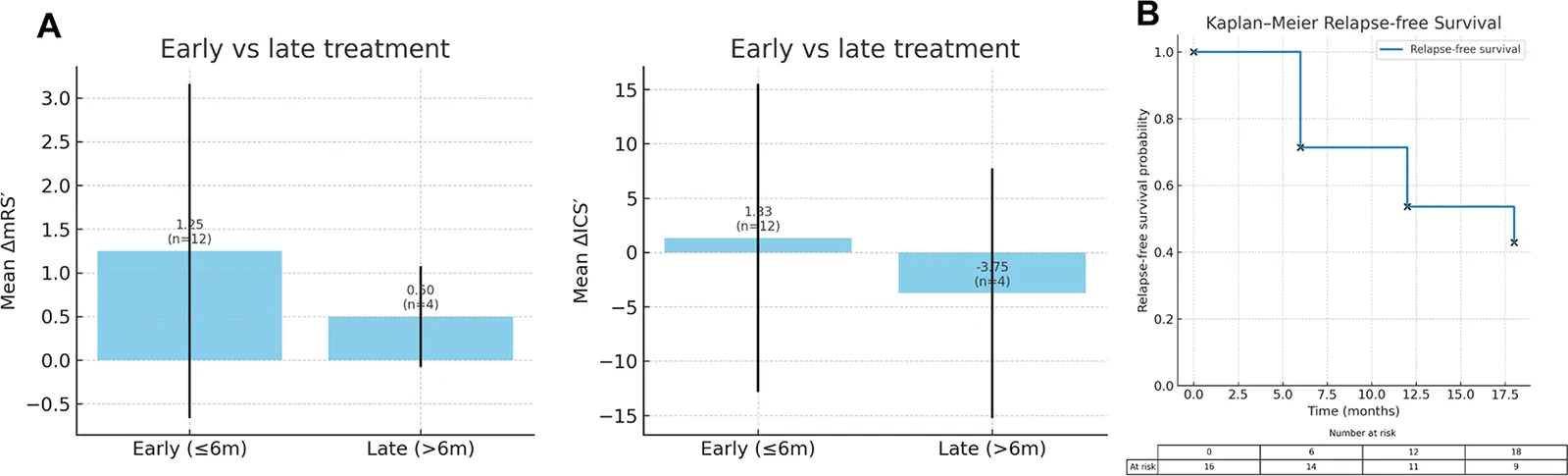
(**A**) Comparison of early (≤ 6 months) versus late (> 6 months) immunotherapy initiation, based on ΔmRS′ and ΔICS′. (**B**) Kaplan–Meier curve of relapse-free survival (RFS). Estimated RFS was 0.714 at 6 months, 0.536 at 12 months, and 0.429 at 18 months. Relapse was defined as recurrent or worsening symptoms after prior improvement during follow-up. Patients without relapse were censored at the last available follow-up

Although the late-treatment group was small, a trend toward greater clinical improvement with the early initiation of immunotherapy was seen (Fig. 6A).

### Relapse-free survival rate

The relapse-free survival (RFS) rate was estimated using the Kaplan–Meier method (*n* = 16). The estimated cumulative RFS probabilities were 0.714 at 6 months, 0.536 at 12 months, and 0.429 at 18 months. The greatest decline in RFS occurred within the first 6 months after discharge, suggesting that relapse events were concentrated in the early post-discharge period. Given the small number of events, these estimates should be interpreted cautiously. (Fig. 6B).

### Association of HLA type with therapeutic efficacy

Among the 16 patients with long-term follow-up, those with HLA-DRB1*10:01 or HLA-DQB1*05:01 alleles (*n* = 11) had a mean ΔmRS′ of 1.18 ± 1.99, compared with 1.00 ± 1.41 in the group without these alleles (*n* = 2). Although this difference was not statistically significant, patients with these alleles tended to achieve greater long-term functional improvements.

All patients with HLA-DQA1*01:05 (*n* = 3) showed clinical improvement, whereas the improvement rate among other patients was 61.5%.

### Computational analysis of HLA-IgLON5 binding

**Fig. 7:**
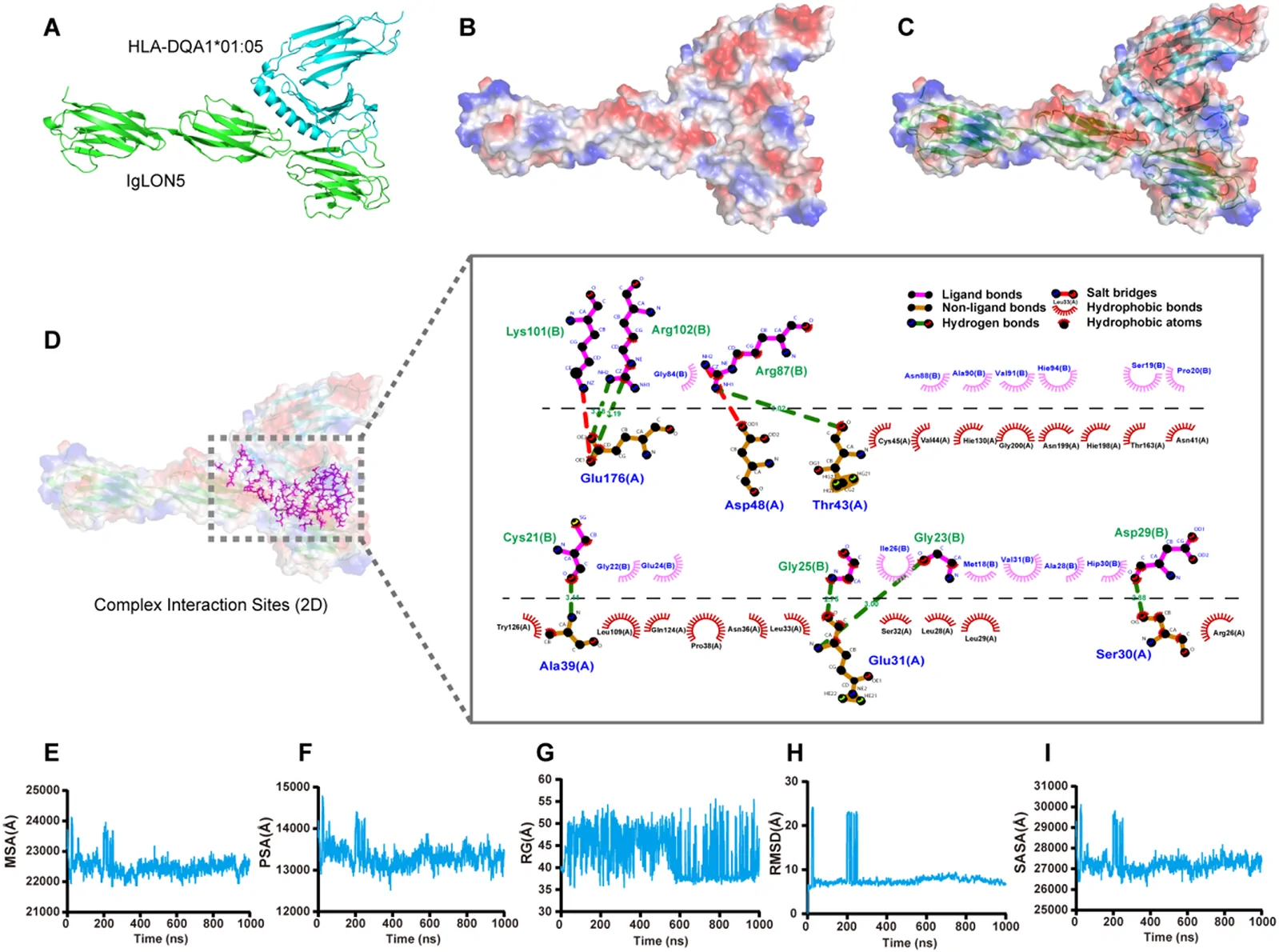
Molecular dynamics (MD) simulation of HLA-DQA1*01:05 and IgLON5. (**A**) Binding pattern of HLA-DQA1*01:05 and the IgLON5 peptide. (**B**) Electrostatic interaction between HLA-DQA1*01:05 and IgLON5. (**C**) Composite diagram of electrostatic interactions at the peptide-binding interface. (**D**) 2D illustration of the interaction sites within the HLA-DQA1*01:05–IgLON5 complex. (**E**–**I**) MD simulation over 1000 ns showing results for polar surface area, molecular surface area, solvent-accessible surface area, radius of gyration, and root-mean-square deviation

Peptide-binding prediction algorithms were employed to investigate the potential interaction between different HLA subtypes and IgLON5. AlphaFold prediction identified an epitope within IgLON5 that is likely recognized by the HLA-DQA1*01:05 subtype (Fig. 7A–C). This epitope, located at the 26th amino acid of the N-terminus, comprises the sequence RGLLS and forms part of the IgLON5 signal peptide. The results indicated that RGLLS is highly likely to bind to HLA-DQA1*01:05, which also closely interacts with the mature IgLON5 peptide (Fig. 7D).

MD simulations were performed to further evaluate the stability and accuracy of this predicted complex. The following five parameters were analyzed over a 1000-ns simulation: polar surface area, molecular surface area, solvent-accessible surface area, gyration radius, and root-mean-square deviation. All parameters progressively stabilized throughout the simulation (Fig. 7E–I). These in silico findings support a possible structural interaction between HLA-DQA1*01:05 and IgLON5-derived peptides, but do not establish a mechanistic or causal relationship.

## Discussion

This retrospective analysis examined 24 patients with anti-IgLON5 disease in 17 centers in China, representing the largest multicenter investigation of this disease in an Eastern population. The mean age at disease onset was 59 years. Most of the 18 patients who underwent immunotherapy demonstrated positive responses. Male patients older than 65 years had poorer prognoses, whereas younger male patients and most female patients responded favorably to immunotherapy. Long-term follow-up (3–60 months) revealed a relapse rate of 25.0%. Additionally, HLA-DQA1*01:05 appeared to be enriched in patients with available HLA typing data.

### Comparison with Western cohorts suggests earlier onset and milder symptoms in Chinese patients

A comparison with previously reported Western cohorts suggests that the Chinese patients in our series may present at a younger age at onset and with milder symptoms. The mean age at onset was approximately 65 years in previous reports from Spain and Germany, whereas in our cohort, it was 59 years [8, 18]. Notably, 33.3% of the patients in our study were younger than 55 years, a demographic rarely observed in earlier studies. The youngest previously reported patient was 40 years old, compared with 33 years old in our series [8].

Additionally, Chinese patients exhibited milder disease severity than Western cohorts, reflected by a mean ICS score of 8.5, markedly lower than the mean scores reported in Western cohorts, such as Barcelona (18) and GENERATE (12)[21]. The higher ICS scores in Western patients likely reflect more severe manifestations, including pronounced sleep disturbances, movement disorders, and bulbar symptoms. The relatively milder presentation in our cohort may be attributed to earlier recognition and diagnosis, with a median diagnostic delay of 2 months compared with approximately 24 months in other populations [25].

Consistent differences were found in comparisons with Western cohorts (Table 4) [16, 25]. Chinese patients exhibited earlier onset, fewer bulbar symptoms, and a higher rate of favorable responses to first-line immunotherapy. These observed differences may reflect population-related factors; however, they should also be interpreted cautiously because of potential differences in follow-up duration, disease severity spectrum, treatment strategies, and referral patterns across the cohorts.

Reports of anti-IgLON5 disease from other Asian populations are limited and mainly restricted to isolated case reports or very small case series. Recent reports from India and Japan described predominantly male patients with sleep disturbances, bulbar and movement symptoms, autonomic involvement, and responsiveness to combined immunotherapy, broadly consistent with our findings [26, 27]. The multicenter cohort in this study provides a more comprehensive characterization of clinical phenotypes, longitudinal outcomes, relapse patterns, and HLA profiles in Chinese patients than these isolated reports.

These observations indicate the need for larger prospective multicenter studies across Asian populations to further clarify regional similarities and differences in clinical phenotypes, HLA types, immunotherapy responsiveness, and long-term prognoses.

**Table 4:** Comparison of clinical characteristics and outcomes between the present Chinese cohort and previously reported Western cohorts

Characteristics	Present Study	Western cohorts
Mean age at onset (y)	59.6	64
Median diagnostic delay, m	2	24
Response to immunotherapy	55.6%	~ 40%
Long-term improvement	71.4%	~ 45–50%
Complete remission	50%	< 20%
Relapse pattern	Often > 1 year post-discharge; clustered at 6–12 months on KM	Not uniformly reported
Mortality	4/24 (16.7%)	44/107 (41.1%)

KM: Kaplan-Meier  Western estimates were mainly derived from Gaig et al. and Grüter et al.; definitions of response, follow-up duration, treatment exposure, and case ascertainment differed across cohorts, limiting direct comparability

### Immunotherapy is effective, particularly with early intervention

Immunotherapy was effective in patients who experienced relapse and was associated with favorable long-term outcomes. In this cohort, 55.6% responded to immunotherapy, higher than the 40% response rate reported in Western populations [16]. Responders had fewer bulbar symptoms than non-responders. This is a notable finding given the high prevalence of bulbar involvement in Western cohorts, which may impede immunotherapy efficacy. The most prominently improved symptoms in descending order among responders were sleep disturbances, bulbar symptoms, and movement disorders. In contrast, the most treatment-refractory manifestations among non-responders were cognitive impairment, followed by sleep disturbances, movement disorders, and bulbar symptoms. These observations highlight the importance of early recognition and intervention.

Patients who initiated immunotherapy within 6 months of symptom onset showed greater functional improvements than those treated later. Although this difference was not statistically significant, a clear trend toward better outcomes was observed, underscoring the value of early treatment.

### Early relapse risk highlights the need for long-term immunotherapy

The relapse rate in the Chinese cohort with long-term follow-up was 25%, a proportion not previously reported in other populations. This may reflect the relatively longer follow-up period in our study than in most existing studies. Most relapses in the patients in our cohort occurred approximately one year after discharge. However, the Kaplan–Meier analysis revealed that RFS rate decreased most sharply within the first 6 months post-discharge, suggesting that this early period represents the most vulnerable window requiring close monitoring and prolonged maintenance therapy.

Relapses were associated with the abrupt discontinuation of treatment or surgical stress and were accompanied by increases in serum antibody titers or seroconversion from negative to positive. The re-initiation of long-term immunotherapy after relapse resulted in marked clinical improvement and sustained stability. These findings support the recommendation that patients with anti-IgLON5 disease receive long-term immunotherapy after discharge, with the gradual tapering of medication and close follow-up for at least 12 to 18 months, particularly during the first year.

### HLA-DQA1*01:05 and its co-occurrence with classical HLA alleles in Chinese patients

Another study focus was to explore whether HLA-DQA1*01:05 co-occurred with classical HLA alleles in Chinese patients with anti-IgLON5 disease. All patients in the cohort with the HLA-DQA1*01:05 allele also carried DRB1*10:01 and DQB1*05:01. HLA-DQA1*01:05 is rare, with a prevalence of only 1% in the southern Chinese population [28] and 1.5% in Caucasians in the United States (Allele Frequency Net Database). Given its rare frequency, the apparent over-representation in this cohort supports possible enrichment in Chinese patients with anti-IgLON5 disease. However, this finding should be interpreted cautiously in the absence of matched controls.

HLA typing of 35 patients in a previous study revealed that among 14 patients with complete HLA sequencing, only three cases (14.3%) carried DQA1*01:05 [16]. However, no association was observed between DQA1*01:05 and DRB1*10:01 or DQB1*05:01 in that study. In contrast, all six patients (100%) positive for DQA1*01:05 in our cohort also carried DRB1*10:01 and DQB1*05:01. Although the sample size was limited, this finding suggests the possible co-occurrence of DRB1*10:01/DQB1*05:01 and DQA1*01:05 in this small Chinese cohort.

MD simulations further supported these observations, suggesting that HLA-DQA1*01:05 and IgLON5 may form a stable binding complex and supporting a possible structural interaction hypothesis. These results are consistent with those in a recent report [29].

Our findings suggest that the genetic background, particularly HLA alleles such as HLA-DRB1*10:01, DQB1*05:01, and DQA1*01:05, may be related to long-term treatment outcomes in patients with anti-IgLON5 disease. Although statistical significance was not achieved due to the limited sample size, the consistent trend toward better functional improvement among patients with these alleles indicates a potential genetic contribution to therapeutic responsiveness and warrants validation in larger cohorts. Additionally, male patients under 65 years and female patients showed superior responses to immunotherapy. Collectively, these genetic associations may provide a biological basis for the distinct clinical phenotype observed in the Chinese cohort, where the interplay between specific HLA haplotypes and the immune response could affect disease presentation, severity, and therapeutic outcomes. This elucidation of population-specific genetic risk factors is an important step toward personalized medicine in autoimmune neurological disorders.

### Anti-IgLON5 disease is frequently misdiagnosed due to atypical and diverse presentations

The median time to diagnose anti-IgLON5 disease in our cohort was 2 months, with some patients experiencing delays of up to 72 months. Notably, 33.3% of patients were initially misdiagnosed. This high rate reflects the diverse and often atypical clinical manifestations of anti-IgLON5 encephalitis, especially in patients with chronic disease. Patients with gradual symptom onset are often misdiagnosed with Parkinson’s disease or sleep disorders, whereas those with acute onset may be mistaken for viral encephalitis, especially if preceded by fever. Individuals presenting with seizures are frequently misdiagnosed with idiopathic epilepsy. These diagnostic challenges highlight the complex interplay between the autoimmune and neurodegenerative aspects of the disease, which hampers accurate diagnosis. Therefore, the careful differentiation of anti-IgLON5 disease from atypical Parkinson’s disease and sleep disorders in clinical practice is critical to ensuring timely and appropriate management.

### Limitations

The findings of this study should be interpreted with caution. Its retrospective multicenter design may have introduced selection and referral biases, ascertainment bias related to chart review, and heterogeneity in assessments and treatments across centers. The small sample size limited statistical power and precluded formal adjustment for potential confounders. In addition, incomplete follow-up and HLA testing in some patients, including eight patients lost to long-term follow-up, may have introduced attrition bias, the impact of which could not be formally assessed. These factors, together with the single-country setting and absence of matched controls, limit the generalizability of the findings to other East Asian populations, mixed-ethnicity cohorts, and non-tertiary care settings, and warrant the cautious interpretation of cross-cohort and HLA-related comparisons.

## Conclusions

This study comprised the largest multicenter cohort of patients with anti-IgLON5 disease in China. Patients in this cohort showed relatively early onset, shorter diagnostic delay, and favorable outcomes after immunotherapy, although cross-cohort comparisons should be interpreted cautiously. The findings also indicate the importance of early immunotherapy within the first year of disease onset, long-term maintenance immunotherapy to reduce relapse risk, and the potential effect of genetics on treatment responses. The results offer novel insight into disease management and underscore the need for population-specific, personalized therapeutic strategies.

## Supplementary Information

Below is the link to the electronic supplementary material.

Supplementary Material 1

## Data Availability

All data relevant to the study are available from the corresponding author upon reasonable request.

## Abbreviations

HLA: Human leukocyte antigen
AE: Autoimmune encephalitis
IgLON5: IgLON family member 5
CNS: Central nervous system
CSF: Cerebrospinal fluid
CBA: Cell-based assay
TBA: Tissue-based assay
IVMP: Intravenous methylprednisolone
IVIG: Intravenous immunoglobulin
ICS: Anti-IgLON5 disease composite score
mRS: The Modified Rankin Scale
MD: Molecular dynamics
MRI: Magnetic resonance imaging
PET: Positron emission tomography
CT: Computed tomography
RFS: Relapse-free survival
