# Supplementary material for: Clinical features, outcome and HLA subtypes in Eastern patients with anti-IgLON5 disease: a multicenter study
Source: Orphanet J Rare Dis. 2026 Jun 12;21:291. doi: 10.1186/s13023-026-04441-z (PMC13487904; doi:10.1186/s13023-026-04441-z)
Supplement: Supplementary file 1 [file 13023_2026_4441_MOESM1_ESM.docx]

**Supplementary Material**

**The 17 medical centers**

Our patients were from 17 different center, including Ruijin Hospital, Huashan Hospital, the Third Affiliated Hospital, Sun Yat-Sen University, Ningbo Medical Centre of Lihuili Hospital, the First Affiliated Hospital of Dalian Medical University, Guangdong Second Provincial General Hospital, the Affiliated Hospital of Hangzhou Normal University, Renji Hospital, the First Affiliated Hospital of Zhengzhou University, Pu'er People's Hospital, the People's Hospital of Guizhou Province, the Second Affiliated Hospital of Guangzhou Medical University, Huizhou Central People's Hospital, the Second Affiliated Hospital of Hainan Medical University, Qilu Hospital, Shandong University, People's Hospital of Hainan Province, and Yuxi City People's Hospital.

**Methods of TBA and immunoprecipitation (IP)**

TBA used a commercially available kit (BioSystems) for tissue sections of the hippocampus and cerebellum of monkeys. The tissues are fixed on glass slides and the operation is carried out according to the instructions. Briefly, CSF without dilution is incubated on the tissue for 2 hours at room temperature. The tissue was then washed in PBS-0.1% Tween-20 three times, incubated for 30 min with fluorescein-conjugated goat anti-human IgG for one hour, washed again, and sealed with a cover glass. Finally, the fluorescence pattern was evaluated using an immunofluorescence microscope. It was validated by co-immunoprecipitation while detecting antibodies. To prepare the antibody-bound magnetic beads, Pierce Protein A/G Magnetic Beads (15 μL) were incubated with 500 μL of blocking buffer, supplemented with 20 μL of healthy control or IgLON5-positive patient sera at room temperature for 4 hours. The beads were washed with 1 mL of TBST twice. Lysates were extracted from HEK293T cells transfected with pcDNA3.1-IgLON5-Flag using the same method used in dot blotting experiments. 400 μL of lysates were subjected to IP by incubating with either control or patient sera antibody-bound magnetic beads at 4℃ overnight. The magnetic beads were washed with TBST five times and then eluted with Laemmli buffer. Finally, western blotting with a commercial IgLON5 antibody (IgLON5 polyclonal antibody, Invitrogen) was used to detect IgLON5.
